# Body Composition of Female Air Force Personnel: A Comparative Study of Aircrew, Airplane, and Helicopter Pilots

**DOI:** 10.3390/ijerph19148640

**Published:** 2022-07-15

**Authors:** Álvaro Bustamante-Sánchez, Pantelis T. Nikolaidis, Vicente Javier Clemente-Suárez

**Affiliations:** 1 Faculty of Sport Sciences, Universidad Europea de Madrid, Tajo Street, s/n, 28670 Madrid, Spain; busta.es@gmail.com (Á.B.-S.); pademil@hotmail.com (P.T.N.); 2 School of Health and Caring Sciences, University of West Attica, 12243 Athens, Greece; 3 Grupo de Investigación en Cultura, Educación y Sociedad, Universidad de la Costa, Barranquilla 080002, Colombia

**Keywords:** women, aircrew, pilots, body fat mass, lean mass, body water

## Abstract

This research aimed to analyze the body composition (BC) of different groups of women aircrew units in the Spanish Air Forces for a better understanding and improvement of their operability. Specifically, 184 female aircrew members were analyzed and classified into specialties (38 airplane pilots, age: 32.8 ± 10.8; 26 helicopter pilots, age: 32.0 ± 9.18; and 120 transport aircrew, age: 36.9 ± 8.18). The women’s BC was analyzed with an InBody720 bioimpedance device previously used in the military population. There were differences in the BC among specialties, although there were similarities between airplane and helicopter pilots. Airplane (24.0% ± 10.4%) and helicopter pilots (22.6 ± 6.32%) had a smaller percentage of body fat mass than transport aircrew (26.3 ± 7.51%), but there was uniformity among groups in skeletal muscle mass and soft lean mass. We found no differences in body water among specialties. Differences in BCs were previously reported for men in the air force, and these results in women showed similarities for different job entry requirements, different training needs, and different occupational behaviors among units in the Air Force. These results help to deepen the previous knowledge of women’s BC standards in military units. Although pilots are primarily responsible for the aircraft, healthy habits should be encouraged to keep fit and improve the performance of all aircrew members both in flight and when they are deployed.

## 1. Introduction

Traditionally, body composition (BC) has been of importance in the military population, because good performance in their duties is closely related to their physical fitness. However, only a few studies have been conducted so far on the BC of air force personnel [1,2,3]. The BC of air force personnel might differ from that of army and navy personnel, reflecting the different physiological demands to which they are subjected [4]. This aspect showed that research findings in army and navy personnel cannot be applied to those in the air force. Moreover, air force personnel should not be considered as a homogeneous population in terms of daily occupational tasks because they are engaged in a wide range of duties, discriminating them in groups such as airplane and helicopter pilots and transport aircrew [5,6]. For instance, pilots present high levels of fat-free mass and low body fat percentages, because a pilot’s G tolerance is related to aero-medical safety [7,8].

BC depends on the aircrew’s job, with airplane pilots being taller, and having more total body water, intracellular water, extracellular water, proteins, minerals, soft lean mass, fat-free mass, and skeletal muscle mass than transport aircrew as well as lower body mass index (BMI) and body fat mass than transport aircrew [1]. So far, studies on the BC of air force personnel have focused on men’s groups, with fewer data reported on women [4,9]. BC is a health-related fitness component that differs between women and men for an average score. This implies that reference data of BC parameters, such as body mass index, fat mass, fat-free mass, and body water—routinely used to evaluate the performance of personnel—should be separately developed for women instead of applying data from men [10].

BMI predicts higher body fat mass in women than in men due to the prevalence of body fat in women and the differences in lean mass for taller men. Knowledge of the BC of air force personnel would be of great practical application considering its relationship with health-related physical fitness and operational performance [11,12]. For instance, a high score of simulated military task performance of an air force unit was related to low fat-free mass [11]. In addition, BC could be informative of eating disorders (anorexia nervosa, bulimia nervosa, and not otherwise specified) in military personnel including the Air Force [13] and correlates with eating behavior [14]. BC has also been shown to be a risk factor of injuries in air force special forces [15] and air force personnel [16], where a large amount of fat mass was related to an increased prevalence of injury. Furthermore, previous research on the BC of female air force personnel relied mostly on anthropometric measures such as BMI, skinfold thickness, and circumferences rather than on more sophisticated assessment methods (e.g., bioelectrical impedance analysis (BIA)) [17,18]. In addition to weight, BMI, body fat percentage, and FFM, BIA can provide information on total body water, intracellular and extracellular water, proteins, minerals, and segmental analysis (i.e., right, and left arm, right and left leg, and trunk) [19,20].

Therefore, this study aimed to (a) analyze body composition differences in women between the types of specialties of aircrews using BIA, (b) better understand their standards, and (c) gather data to improve their preparation for the hazards they must face during their duty. We hypothesized that (i) there would be differences in body composition among specialties, (ii) airplane and helicopter pilots would have a lower percentage of body fat, more soft lean mass, and more skeletal muscle mass than transport aircrew, and (iii) airplane and helicopter pilots would have more body water (total, intracellular and extracellular) than transport aircrew.

## 2. Materials and Methods

### 2.1. Subjects

A cross-sectional experimental design was conducted to analyze body composition differences in military women depending on their specialty in the Air Force. Approximately 184 Caucasian female aircrew members were analyzed and classified according to their specialty (38 airplane pilots, age: M = 32.8, SD = 10.8; 26 helicopter pilots, age: M = 32.0, SD = 9.18; and 120 transport aircrew, age: M = 36.9, SD = 8.18) in the Spanish Army with a “fit” medical condition according to the ministerial order 23/2011, which requires a periodic aeromedical training that agrees with the STANAG 3114 “Aeromedical Training of Flight Personnel” (NATO regulations). The procedures of this research were explained to all the participants who then gave their voluntary written informed consent under the Declaration of Helsinki. The design of this study was created and approved by the Medical Service of the Aerospace Medicine Instruction Centre of Spanish Air Forces and the University Ethic Committee (CIPI/21/072).

### 2.2. Procedure

To assess body composition, previous methods were followed [1,21,22,23] in which the individuals stood barefoot on the feet electrodes of the body impedance base with their lower limbs not touching their trunk. The bioimpedance instrument had four feet-electrodes. Before the test, the skin was cleaned and dried. Individuals also had to grip the palm and thumb electrodes. The bioimpedance instrument was calibrated before the evaluation, and all the electrodes were cleaned with alcohol before each test. Participants were asked not to drink alcohol and not to perform vigorous exercise for 24 h prior to the test. To control hydration status, the measurements were recorded in the morning after overnight fasting, similar to previous research with bioelectrical impedance [1]. The measurements started at 8 a.m. and lasted until 9 a.m.

### 2.3. Materials

A bioimpedance analyzer (InBody 720, Biospace Co. Ltd., Seoul, Korea) was used for body mass measurement (to the nearest 0.1 kg) and body height (to the nearest 0.1 cm). InBody 720 is an impedance body composition device that uses electrodes to measure resistance at different frequencies from 1 kHz to 1 MHz and reactance at frequencies from 5 to 250 kHz. It measures body composition in five segments (right arm, left arm, trunk, right leg, and left leg). Bioelectrical impedance analysis (BIA) has been proven to be a reliable measure of body composition when compared to the gold standard body composition measurement (dual-energy X-ray absorptiometry (DXA)) for a wide range of normal and overweight populations [24,25,26] and military samples [27].

Data were electronically imported to Excel using Lookin’Body 3.0 software (InBody 720, Biospace Co. Ltd., Seoul, Korea). The following parameters were analyzed: (i) body mass (kg), (ii) height (cm), (iii) body mass index (BMI) (kg/m^2^), (iv) total body water (TBW) (kg) (including left and right arms, trunk, and left and right legs), (v) intracellular water (ICW) (l) (including left and right arms, trunk, and left and right legs), (vi) extracellular water (ECW) (l) (including left and right arms, trunk, and left and right legs), (vii) proteins (kg), (viii) minerals (kg), (ix) body fat mass (BFM) (kg) (including left and right arms, trunk, and left and right legs, in kg and %), (x) percentage of body fat (PBF) (%), (xi) soft lean mass (SLM) (kg), (xii) fat-free mass (FFM) (kg) (including left and right arms, trunk, and left and right legs, in kg and %), and (xiii) skeletal muscle mass (SMM) (kg).

### 2.4. Statistical Analysis

The SPSS statistical package (version 21.0; SPSS, Inc. Chicago, IL, USA) was used to analyze the data. Normality assumptions were checked with the Kolmogorov–Smirnov test. The Kruskal–Wallis test was used to compare the type of work on each parameter. The Mann–Whitney U test, together with a Bonferroni post hoc test, was used to analyze pairwise comparisons. The effect size was assessed by the eta squared value (η^2^, the ratio of the sum of squares for the effect divided by the total sum of squares) and calculated through SPSS. The level of significance for all the comparisons was set at *p* ≤ 0.05.

## 3. Results

The results are reported with their mean and standard deviation. There were no significant differences in age among groups. Table 1 shows the results of body composition. There was a significant difference between specialties in the body mass results. Helicopter pilots (M = 60.1 kg) were lighter (*p* < 0.05) than transport aircrew (M = 63.4 kg). There was a significant effect of specialty on the BMI results. Helicopter pilots (M = 21.5 kg/m^2^) had a lower BMI (*p* < 0.05) than transport aircrew (M = 23.1 kg/m^2^). There was a significant difference between specialties in the body fat mass results. Airplane pilots (M = 15.8 kg) and helicopter pilots (M = 13.8 kg) had less body fat mass (*p* < 0.05) than transport aircrew (M = 17.2 kg). There was a significant difference between specialties in the percentage of body fat mass results. Airplane pilots (M = 24.0%) and helicopter pilots (M = 22.6%) had a lower percentage of body fat mass (*p* < 0.05) than transport aircrew (M = 26.3%). No significant differences were found among groups for the height, TBW, ICW, proteins, minerals, SLM, FFM, or SMM.

Table 2 shows the results of FFM and BFM by body segments. There was a significant difference between specialties in the body fat mass of the right arm, left arm, trunk, right leg, and left leg. Airplane pilots and helicopter pilots had less body fat mass (*p* < 0.05) than transport aircrew in all cases. No significant differences were found for the FFM values by segments.

Table 3 shows the results of body water (total, intracellular, and extracellular) by body segment. No significant differences were found for each different specialty in these variables.

## 4. Discussion

This study aimed to analyze body composition differences among various specialties in women military aircrews. The first hypothesis was accepted because there were differences in body composition between specialties, although there were similarities between airplane and helicopter pilots. The second hypothesis was partially accepted because airplane and helicopter pilots had a lower percentage of body fat mass, but there was uniformity among groups in skeletal muscle mass and soft lean mass. The third hypothesis was rejected because there were no differences in body water among specialties.

The results of the analysis of the height showed equivalences among groups, which differs from the results previous studies that reported differences between pilots and transport aircrew [1], probably due to the different standards of diverse military occupational specialties. These standards are based on human limitations for using specific military equipment that has a minimum height requirement [10]. Although height is used to modulate weight, and both measurements are normally related, we found dissimilarities in weight and BMI between helicopter pilots and transport aircrew. When we analyzed the results for body fat mass and the percentage of body fat, which provide further insights than just body mass and BMI assessment [28,29], we confirmed that the pilots’ groups were predominantly fitter than the transport aircrew. Differences between pilots and transport aircrew in BMI were previously reported for men in air forces [1], and the results of the present study in women showed a continuous tendency toward different requirements to enter the air force, different training needs, and different occupational behaviors among units [30,31].

BFM and PBF usually show an inverse relationship with SLM, FFM, and SMM, as was reported in male aircrews [1]. Despite the dissimilarities in fat-related values, we did not find differences between groups in soft lean mass or muscle mass for women, which could be related to the increased body fat percentage in women or because of a higher influence of lean mass on the BMI results for men due to their longer skeletal muscle proportions [10]. This should be better explored in future research, although it highlights the need to study and develop body composition standards for military women, to avoid health risks and a decrease in their occupational performance because of too stringent requirements based on male standards [32,33]. Skeletal muscles are built in protein and water, so not finding differences in these items was coherent with results of previous studies in athletes [22,28].

Similarly, there were similitudes in the values of fat-free mass by body segment, but there were differences for all the segments analyzed in body fat mass. The greater body fat mass in left and right legs and arms, and the trunk, was previously reported for male transport aircrew compared with helicopter and airplane pilots [1], and should be better explored after training interventions to assess if regional changes in body composition are coherent with the training goals and performance needs [34,35,36].

No differences were found in body water values (total, intracellular, and extracellular), although these results could be due to the small sample size for the pilots’ groups. Apart from the relationship among skeletal muscle mass, protein, and body water, the association between body water and performance has been studied in athletes, where motor skills [37,38,39] and attention [40] were positively influenced by higher values of TBW and ICW. Similarly, body water values were found higher in male airplane pilots (whose cognitive and physical performance is crucial to maintaining the requirements of a safe flight) compared with transport aircrew, as in previous research [1]. The lower skeletal muscle mass in women, compared with men, and its relationship with body water could explain these differences, although further research together with hydration and dehydration interventions could help to clarify this.

### Study Limitations

One of the limitations of this study, due to the restricted material and human resources, was the impossibility of evaluating the body perimeters (e.g., waist and hip circumferences) of participants to have a complete anthropometric analysis [33]. Due to the accessibility of women aircrew, we included all participants who were available to explore the characteristics of these units. Future research could consider power calculations to choose the sample sizes. These data could be applied in the future for risk management or education, but we did not use them to assess risk management through the comparison of accident rate and body composition parameters. Another limitation was the inability to control physical activity and nutrition habits of participants, variables that could have a direct impact on BC. Future research should consider these issues.

## 5. Practical Applications

- There is still no consensus about the best body composition (BC) standards for the wide range of age, job, and duties, especially in women.
- Although skeletal muscle mass and soft lean mass values were uniform among specialties, aircraft pilots had a lower body fat percentage than transport aircrew.
- These results could help to deepen our knowledge of women’s BC standards among military units with different entry requirements and distinct training protocols.

## 6. Conclusions

Female aircrew BC values were different depending on each specialty. Airplane and helicopter pilots had a lower percentage of body fat mass than transport aircrew, but there were similarities among groups in skeletal muscle mass and soft lean mass. We found no differences in body water among specialties. Differences in BC were previously reported for men in air forces, and these results in women showed similarities for different job entry requirements (with the most difficult physical tests for pilots) and different training needs (with regular endurance and resistance training for pilots), among units in the air force. These results help to deepen the knowledge of women’s BC standards in military units. Pilots are primarily responsible for aircraft, and healthy habits (focused on adequate nutrition and specific training) should be encouraged to maintain their body composition levels among the standards for each unit, both in flight and when they are deployed. These results can lead to specific intervention programs aimed at maintaining a percentage of high muscle mass and low fat and a diet that helps achieve these goals.

## Figures and Tables

**Table 1:** Results of body composition variables by specialty.

	Airplane Pilots		Helicopter Pilots		Transport Aircrew		Specialty Effect		
	M	SD	M	SD	M	SD	H (2)	*p*	η^2^
Height (cm)	165.1	4.84	166.7	5.87	165.6	4.95	2.079	0.364	0.008
Body Mass (kg)	61.2	12.5	60.1 *	10.7	63.4	10.1	6.049	0.049	0.015
BMI (kg/m^2^)	22.4	4.15	21.5 *	3.08	23.1	3.45	8.939	0.011	0.025
TBW (l)	33.2	3.41	33.8	5.87	33.7	3.67	1.482	0.477	0.003
ICW (l)	20.7	2.14	21.0	3.74	21.0	2.31	1.424	0.491	0.002
ECW (l)	12.5	1.29	12.7	2.12	12.7	1.37	1.732	0.421	0.006
Proteins (kg)	8.96	0.91	9.09	1.62	9.08	1.00	1.486	0.476	0.002
Minerals (kg)	3.24	0.34	3.36	0.51	3.35	0.37	3.486	0.175	0.015
BFM (kg)	15.8 *	10.3	13.8 *	5.21	17.2	7.58	10.011	0.007	0.023
PBF (%)	24.0 *	10.4	22.6 *	6.32	26.3	7.51	8.964	0.011	0.030
SLM (kg)	42.7	4.38	43.4	7.59	43.4	4.73	1.483	0.476	0.003
FFM (kg)	45.4	4.65	46.2	7.99	46.2	5.04	1.552	0.460	0.003
SMM (kg)	25.0	2.79	25.4	4.89	25.3	3.28	1.377	0.502	0.002

M: mean. SD: standard deviation. H: Kruskal–Wallis H-test. η^2^: partial eta squared. BMI: body mass index. TBW: total body water. ICW: intracellular water. ECW: extracellular water. BFM: body fat mass. PBF: percentage of body fat. SLM: soft lean mass. FFM: fat-free mass. SMM: skeletal muscle mass. * Differences with the transport aircrew group (*p* < 0.05).

**Table 2:** Results of fat-free ass and body fat mass by body segment.

	Airplane Pilots		Helicopter Pilots		Transport Aircrew		Specialty Effect		
	M	SD	M	SD	M	SD	H (2)	*p*	η^2^
FFM Right Arm (kg)	2.27	0.37	2.30	0.63	2.31	0.38	1.469	0.480	0.001
FFM Left Arm (kg)	2.23	0.35	2.27	0.64	2.27	0.38	1.847	0.397	0.002
FFM Trunk (kg)	19.9	2.22	20.1	3.75	20.2	2.31	1.427	0.490	0.001
FFM Right Leg (kg)	7.04	0.81	7.02	1.18	7.05	0.80	0.747	0.688	0.000
FFM Left Leg (kg)	7.02	0.78	7.00	1.18	7.03	0.80	0.643	0.725	0.000
BFM Right Arm (kg)	1.10 *	0.94	0.85 *	0.39	1.17	0.74	9.819	0.007	0.020
BFM Left Arm (kg)	1.11 *	0.95	0.88 *	0.39	1.19	0.73	8.870	0.012	0.019
BFM Trunk (kg)	7.55 *	5.22	6.80 *	3.12	8.53	3.96	9.449	0.009	0.024
BFM Right Leg (kg)	2.51 *	1.52	2.12 *	0.61	2.65	1.02	11.326	0.003	0.026
BFM Left Leg (kg)	2.50 *	1.51	2.11 *	0.62	2.63	1.02	10.868	0.004	0.026

M: mean. SD: standard deviation. H: Kruskal–Wallis H-test. η^2^: partial eta squared. FFM: fat-free mass. BFM: body fat mass. * Differences with the transport aircrew group (*p* < 0.05).

**Table 3:** Results of body water by body segment.

	Airplane Pilots		Helicopter Pilots		Transport Aircrew		Specialty Effect		
	M	SD	M	SD	M	SD	H (2)	*p*	η^2^
TBW Right Arm (l)	1.76	0.28	1.79	0.49	1.79	0.30	1.532	0.465	0.001
TBW Left Arm (l)	1.73	0.27	1.76	0.50	1.77	0.30	1.857	0.395	0.002
TBW Trunk (l)	15.5	1.72	15.7	2.89	15.7	1.80	1.401	0.496	0.001
TBW Right Leg (l)	5.47	0.63	5.46	0.91	5.48	0.62	0.704	0.717	0.000
TBW Left Leg (l)	5.46	0.61	5.44	0.91	5.47	0.62	0.664	0.717	0.000
ICW Right Arm (l)	1.10	0.17	1.12	0.30	1.12	0.18	1.298	0.523	0.001
ICW Left Arm (l)	1.08	0.17	1.10	0.30	1.10	0.18	1.606	0.448	0.001
ICW Trunk (l)	9.68	1.09	9.75	1.86	9.75	1.13	1.305	0.521	0.000
ICW Right Leg (l)	3.42	0.39	3.40	0.58	3.42	0.39	0.943	0.624	0.000
ICW Left Leg (l)	3.41	0.37	3.39	0.58	3.40	0.38	0.850	0.654	0.000
ECW Right Arm (l)	0.66	0.10	0.67	0.18	0.67	0.11	2.065	0.356	0.002
ECW Left Arm (l)	0.64	0.10	0.66	0.19	0.66	0.11	2.392	0.302	0.003
ECW Trunk (l)	5.85	0.64	5.94	1.04	5.96	0.67	1.828	0.401	0.004
ECW Right Leg (l)	2.04	0.24	2.05	0.33	2.06	0.24	0.559	0.756	0.001
ECW Left Leg (l)	2.05	0.24	2.05	0.33	2.07	0.24	0.596	0.742	0.001

M: mean. SD: standard deviation. H: Kruskal–Wallis H-test. η^2^: partial eta squared. TBW: total body water. ICW: intracellular water. ECW: extracellular water.

## Data Availability

Not applicable.
